# Dynamic, Simultaneous Concentration Mapping of Multiple MRI Contrast Agents with Dual Contrast - Magnetic Resonance Fingerprinting

**DOI:** 10.1038/s41598-019-56531-7

**Published:** 2019-12-27

**Authors:** Christian E. Anderson, Mette Johansen, Bernadette O. Erokwu, He Hu, Yuning Gu, Yifan Zhang, Michael Kavran, Jason Vincent, Mitchell L. Drumm, Mark A. Griswold, Nicole F. Steinmetz, Ming Li, Heather Clark, Rebecca J. Darrah, Xin Yu, Susann M. Brady-Kalnay, Chris A. Flask

**Affiliations:** 1 Department of Radiology, Case Western Reserve University, Cleveland, OH USA; 2 Department of Biomedical Engineering, Case Western Reserve University, Cleveland, OH USA; 3 Department of Molecular Biology and Microbiology, Case Western Reserve University, Cleveland, OH USA; 4 Department of NanoEngineering, University of California-San Diego, La Jolla, CA USA; 5 Department of Genetics and Genome Sciences, Case Western Reserve University, Cleveland, OH USA; 6 Department of Pediatrics, Case Western Reserve University, Cleveland, OH USA; 7 Department of Radiology, University of California-San Diego, La Jolla, CA USA; 8 Moores Cancer Center, University of California-San Diego, La Jolla, CA USA; 9 Department of Population and Quantitative Health Sciences, Case Western Reserve University, Cleveland, OH USA; 10 Department of Bioengineering, Northeastern University, Boston, MA USA; 11 Department of Chemistry and Chemical Biology, Northeastern University, Boston, MA USA; 12 Institute of Systems Bioanalysis and Chemical Imaging, Northeastern University, Boston, MA USA; 13 Francis Payne Bolton School of Nursing, Case Western Reserve University, Cleveland, OH USA; 14 Department of Physiology and Biophysics, Case Western Reserve University, Cleveland, OH USA; 15 Department of Neurosciences, Case Western Reserve University, Cleveland, OH USA

**Keywords:** Cancer imaging, Preclinical research, Biomedical engineering

## Abstract

Synchronous assessment of multiple MRI contrast agents in a single scanning session would provide a new “multi-color” imaging capability similar to fluorescence imaging but with high spatiotemporal resolution and unlimited imaging depth. This multi-agent MRI technology would enable a whole new class of basic science and clinical MRI experiments that simultaneously explore multiple physiologic/molecular events *in vivo*. Unfortunately, conventional MRI acquisition techniques are only capable of detecting and quantifying one paramagnetic MRI contrast agent at a time. Herein, the Dual Contrast – Magnetic Resonance Fingerprinting (DC-MRF) methodology was extended for *in vivo* application and evaluated by simultaneously and dynamically mapping the intra-tumoral concentration of two MRI contrast agents (Gd-BOPTA and Dy-DOTA-azide) in a mouse glioma model. Co-registered gadolinium and dysprosium concentration maps were generated with sub-millimeter spatial resolution and acquired dynamically with just over 2-minute temporal resolution. Mean tumor Gd and Dy concentration measurements from both single agent and dual agent DC-MRF studies demonstrated significant correlations with *ex vivo* mass spectrometry elemental analyses. This initial *in vivo* study demonstrates the potential for DC-MRF to provide a useful dual-agent MRI platform.

## Introduction

Intravenously administered paramagnetic MRI contrast agents are a hallmark of radiological practice. These MRI contrast agents are intended to preferentially accumulate in pathologic tissue enabling improved disease detection through alterations in the magnetic properties of the local tissue. Specifically, paramagnetic MRI contrast agents (e.g., gadolinium chelates^1^) shorten the T_1_ and T_2_ magnetic relaxation time constants of the local tissue. While these paramagnetic MRI contrast agents provide improved disease detection individually, the combination of two or more contrast agents in a single MRI scan could provide additional diagnostic and prognostic information. As an example, two different MRI contrast agents could be co-administered to simultaneously assess a tumor’s vasculature (e.g., a large, macromolecular blood-pool MRI contrast agent^2^) as well as the tumor’s vascular permeability (e.g., a smaller extravascular contrast agent^3^) to provide a more comprehensive assessment of the tumor’s vascular network. The continued expansion of the portfolio of highly-specific molecular MRI contrast agents will provide numerous additional multi-agent imaging opportunities. In this situation, a molecular “theranostic” MRI contrast agent could be used to monitor the delivery of a therapeutic molecule to the disease site^4^, while a second molecular MRI contrast agent could be used to assess therapeutic efficacy^5^ providing a combined simultaneous voxelwise assessment of therapeutic delivery and response. Alternatively, two molecular MRI contrast agents could be combined to assess both gene expression (e.g., reporter genes^6,7^) and the downstream effects of the gene’s function such as neurotransmitter release^8^, ion concentration^9^, protein production^10^, or enzymatic activity^11^. In this way a “two-color” MRI method could be used in a similar fashion to how multi-agent imaging studies are routinely conducted in basic science fluorescence imaging experiments^12^. However, “two-color” MRI would offer the additional advantages of high spatial resolution, 3D imaging capabilities, and unlimited imaging depth necessary for non-invasive human imaging studies.

Despite the variety of potential clinical and basic science applications, the use of multiple paramagnetic MRI contrast agents has not yet been fully realized. The primary difficulty in detecting two contrast agents at the same time is that each agent results in a concentration-dependent reduction in both the T_1_ and T_2_ relaxation time constants of the tissue according to the well-established relaxation equations^13^:

1a $$1/{{\rm{T}}}_{1}=1/{{\rm{T}}}_{10}+{{\rm{r}}}_{1{\rm{A}}}\times [{\rm{A}}]$$

1b $$1/{{\rm{T}}}_{2}=1/{{\rm{T}}}_{20}+{{\rm{r}}}_{2{\rm{A}}}\times [{\rm{A}}]$$

where [A] is the concentration of contrast agent A; T_10_, T_20_, T_1_, and T_2_ are the pre-contrast and post-contrast relaxation time constants of the tissue, respectively; and r_1A_ and r_2A_ are the magnetic relaxivities of contrast agent A. Using this model, independent quantification of two different contrast agents (e.g., a gadolinium chelate and an iron oxide agent) following simultaneous injection is difficult because these agents can both individually cause substantial T_1_ and T_2_ changes.

Recent *in vitro* work developed a pathway towards detecting multiple contrast agents by simultaneously assessing both the T_1_ and T_2_ relaxation time constants and proposing a new multi-agent relaxation model^14^:

2a $$1/{{\rm{T}}}_{1}=1/{{\rm{T}}}_{10}+{{\rm{r}}}_{1{\rm{A}}}\times [{\rm{A}}]+{{\rm{r}}}_{1{\rm{B}}}\times [{\rm{B}}]$$

2b $$1/{{\rm{T}}}_{2}=1/{{\rm{T}}}_{20}+{{\rm{r}}}_{2{\rm{A}}}\times [{\rm{A}}]+{{\rm{r}}}_{2{\rm{B}}}\times [{\rm{B}}]$$

With this model, it was shown that simultaneous assessment of T_1_ and T_2_ provided by the Magnetic Resonance Fingerprinting methodology could be used to directly solve Eqs. 2a and 2b in order to calculate voxelwise concentration maps for agents A and B. Together, this multi-agent model and MRF acquisition strategy comprised a new methodology, termed Dual Contrast – Magnetic Resonance Fingerprinting (DC-MRF). However, the practical capability of DC-MRF to detect two MRI contrast agents *in vivo* remained undetermined. Therefore, the goal of this study is to further develop and evaluate the DC-MRF methodology to dynamically measure the concentration of two MRI contrast agents *in vivo*.

## Results

For this initial *in vivo* study, the DC-MRF methodology was used to simultaneously detect a gadolinium chelate (Gd-BOPTA) and a dysprosium chelate (Dy-DOTA-azide) in a mouse glioma model. These two contrast agents were selected on the basis of the following rationale: (1) both agents have been previously studied in animal models^15,16^; (2) both agents have similar molecular weights and should therefore be expected to have similar tumor pharmacokinetics^15^; and (3) these two contrast agents have different magnetic relaxivity ratios (r_2_/r_1_, Supplementary Fig. 1) enabling the multi-agent relaxation model (Eqs. 2a and 2b) to be solved. The LN-229 glioma model was selected for this study because it was previously shown to exhibit substantial contrast agent accumulation during a dynamic imaging session^17^.

### *In vivo* magnetic relaxivity assessments

**Figure 1:**
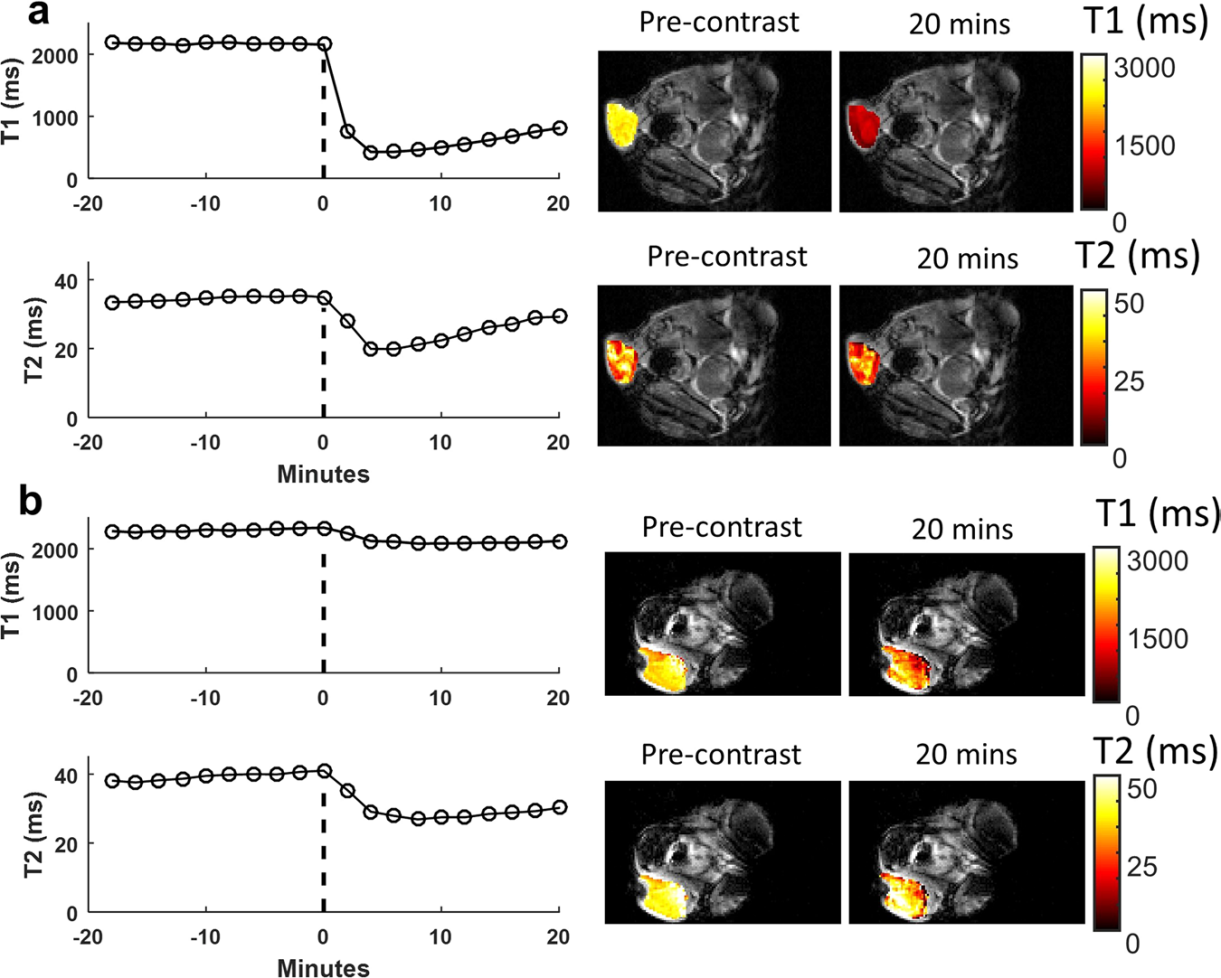
Dynamic contrast enhanced T_1_ and T_2_ measurements. Representative single agent MRF-based T_1_ and T_2_ relaxation time constant curves and maps are shown for (**a**) Gd-BOPTA (0.4 mmol/kg) or (**b**) Dy-DOTA-azide (0.5 mmol/kg) MRI contrast agents. The vertical dotted black line indicates the time of contrast agent bolus injection following ten successive pre-contrast scans. The pre-contrast T_1_ and T_2_ maps shown are from the MRF scan acquired immediately prior to injection (0 minutes). The final 20-minute post-contrast MRF scan was obtained immediately prior to tumor excision. Maps are shown as T_1_ or T_2_ tumor maps superimposed on a reference anatomical image.

The first step in validating the DC-MRF methodology was to assess the *in vivo* magnetic relaxivities (r_1_ and r_2_) of the Gd-BOPTA and Dy-DOTA-azide contrast agents. To accomplish this, either Gd-BOPTA or Dy-DOTA-azide was injected as a single agent over a range of doses (0.1–0.4 mmol/kg for Gd-BOPTA, n = 14; 0.3–1.3 mmol/kg for Dy-DOTA-azide, n = 17) during serially acquired dynamic MRF scans to assess the T_1_ and T_2_ relaxation time constants before and after contrast agent administration. In addition, a single animal was injected with saline only to act as a sham control (n = 1). The dynamic MRF scans resulted in 20 sets of T_1_ and T_2_ relaxation time constant maps (10 pre-contrast T_1_ and T_2_ maps; 10 post-contrast T_1_ and T_2_ maps) acquired every ~2 minutes. A region-of-interest (ROI) analysis generated mean intra-tumoral T_1_ and T_2_ values for each of the 20 MRF scans. Figure 1 shows example MRF-based dynamic T_1_ and T_2_ curves and representative maps for both a Gd-BOPTA (injected dose = 0.4 mmol/kg, Fig. 1a) and a Dy-DOTA-azide (injected dose = 0.5 mmol/kg, Fig. 1b) experiment. The pre-contrast T_1_ and T_2_ maps shown in Fig. 1 were from Scan 10 acquired immediately prior to contrast agent injection while the post-contrast maps were from the final dynamic MRF acquisition (Scan 20). These data show that the serial MRF acquisitions can dynamically detect reductions in tumor T_1_ and T_2_ relaxation time constants resulting from the accumulation of each contrast agent within the tumor.

**Figure 2:**
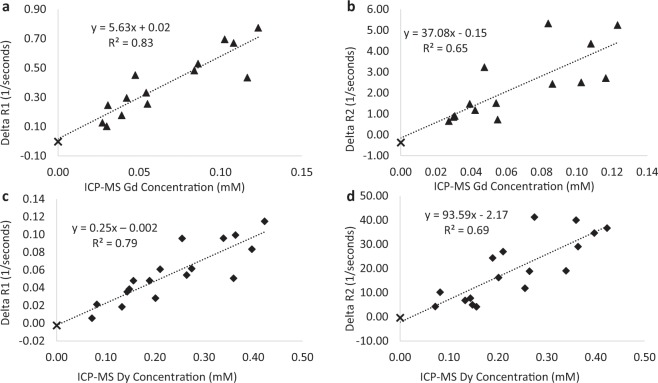
*In vivo* MRI contrast agent relaxivity estimates. T_1_ enhancement (ΔR_1_ = 1/T_1_–1/T_10_) and T_2_ enhancement (ΔR_2_ = 1/T_2_–1/T_20_) are plotted against the corresponding ICP-MS measurement of tumor (**a**,**b**) Gd (triangles, n = 14) or (**c**,**d**) Dy (diamonds, n = 17) concentration. A single sham control is also included (black ‘x’, n = 1). For this calibration step, analyses were performed using only the experiments where the agent of interest was present in the sample. T_10,20_ were taken as the average of the 10 pre-contrast MRF maps, and T_1,2_ were from the final (20 minutes post-contrast) MRF maps. A least-squares regression resulted in significant correlations for all experiments (p < 0.001). The slope of the linear fit in each plot is the *in vivo* magnetic relaxivity ((**a**,**c**) r_1_ and (**b**,**d**) r_2_) of each contrast agent.

Following acquisition of the final MRF map, each animal was immediately euthanized and the tumors were resected for elemental analysis by inductively coupled plasma-mass spectrometry (ICP-MS) to measure the intra-tumoral Gd and Dy concentration. Differences between the pre-contrast and post-contrast tumor T_1_ and T_2_ relaxation time constants (ΔR_1_ and ΔR_2_) were then used with the corresponding ICP-MS measurements of concentration to estimate the *in vivo* magnetic relaxivities of the Gd-BOPTA and Dy-DOTA-azide contrast agents using Eqs. 1a and 1b (Fig. 2). A linear regression of ΔR_1_ (1/T_1_–1/T_10_) and ΔR_2_ (1/T_2_–1/T_20_) with Gd concentration (n = 15) resulted in Gd-BOPTA relaxivity estimates of: r_1_ = 5.63 L∙mmol^−1^∙sec^−1^ (Fig. 2a, R^2^ = 0.83, p < 1e^−5^) and r_2_ = 37.08 L∙mmol^−1^∙sec^−1^ (Fig. 2b, R^2^ = 0.65, p < 0.001). A similar regression analysis for the Dy-DOTA-azide experiments (n = 18) resulted in magnetic relaxivity estimates of: r_1_ = 0.25 L∙mmol^−1^∙sec^−1^ (Fig. 2c, R^2^ = 0.79, p < 1e^−6^) and r_2_ = 93.59 L∙mmol^−1^∙sec^−1^ (Fig. 2d, R^2^ = 0.69, p < 0.0001). It is important to note the resulting r_2_/r_1_ ratios for Gd-BOPTA (r_2_/r_1_ = 6.59) and Dy-DOTA-azide (r_2_/r_1_ = 374) were distinct and suggested that Eqs. 2a and 2b could be solved for the individual concentrations following simultaneous administration.

### *In vivo* dual agent MRF assessments

**Figure 3:**
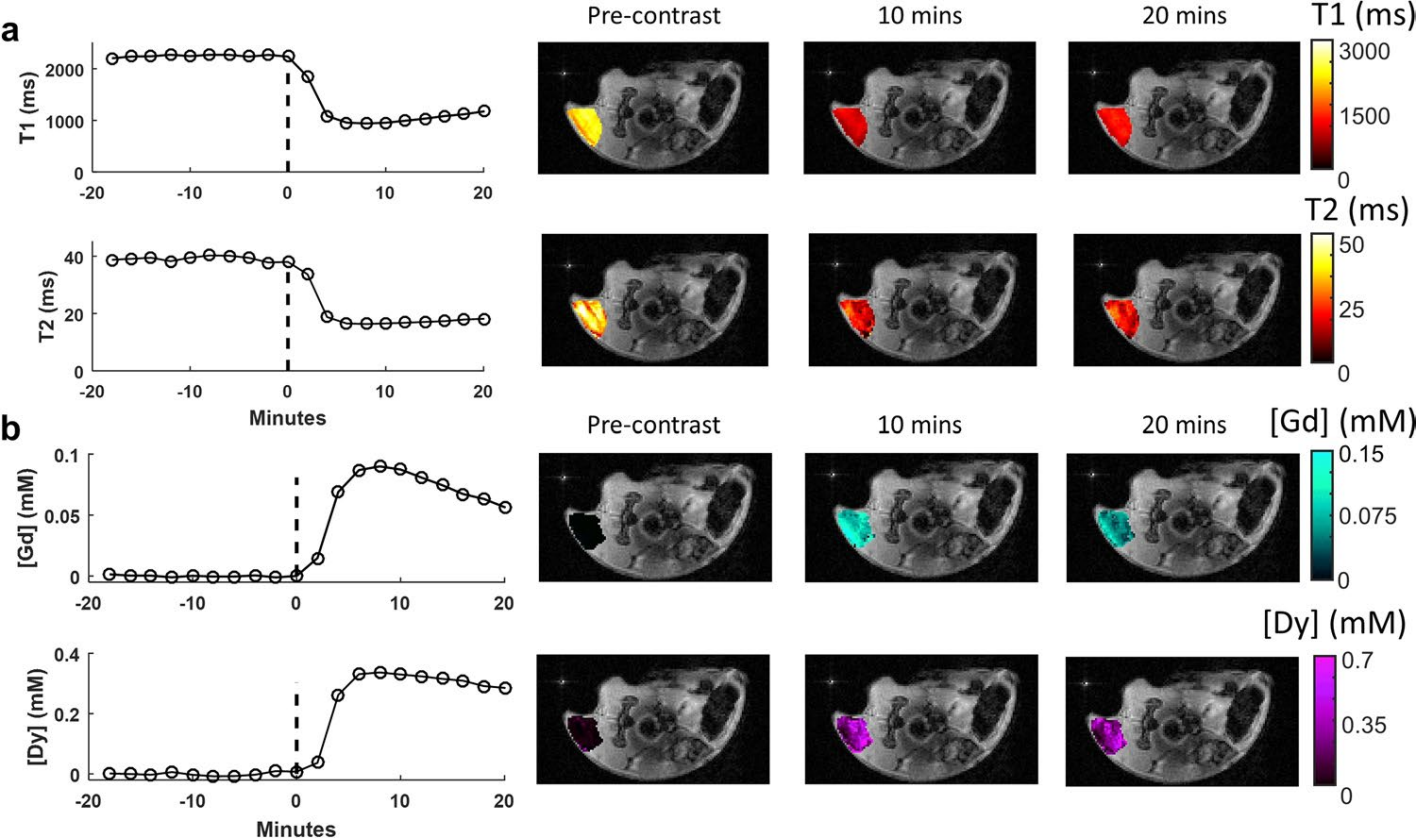
Non-invasive MRI-based dual agent concentration measurements. (**a**) Dynamic MRF-based T_1_ and T_2_ curves and maps following simultaneous administration of Gd-BOPTA (0.15 mmol/kg) and Dy-DOTA-azide (1.1 mmol/kg). Visible reductions of the tumor T_1_ and T_2_ relaxation time constants were observed in both the curves and maps due to tumor uptake of the two contrast agents. (**b**) Corresponding DC-MRF Gd and Dy concentration curves obtained from the multi-agent relaxation model (Eqs. 2a and 2b) and estimated *in vivo* relaxivities (from Fig. 2) show visible increases in tumor Gd and Dy concentration.

Using the same dynamic MRF acquisition, dual agent MRF experiments were conducted to assess the ability of the DC-MRF method to accurately measure the individual concentrations of the Gd and Dy contrast agents following simultaneous administration. Different combinations of Gd-BOPTA and Dy-DOTA-azide were injected as a mixture over a range of doses (Gd 0.15–0.30 mmol/kg and Dy 0.30–1.10 mmol/kg, n = 8) with representative mean tumor T_1_ and T_2_ curves and associated pre-contrast and post-contrast maps (Gd-BOPTA dose: 0.15 mmol/kg; Dy-DOTA-azide dose: 1.1 mmol/kg) shown in Fig. 3a. The *in vivo* magnetic relaxivities estimated from the single agent studies described above were then used along with the DC-MRF multi-agent relaxation model (Eqs. 2a and 2b) to calculate maps of tumor Gd and Dy concentration for each dynamic MRF scan. Figure 3b shows both Gd and Dy concentration versus time curves for the same mouse in Fig. 3a. As expected, the pre-contrast Gd and Dy concentration maps and curves (from Scan 10, 0 minute timepoint) show little or no Gd or Dy in the tumor prior to administration of the agents. In comparison, the post-contrast Gd and Dy concentration maps and curves (Scan 15, 10-minute timepoint; Scan 20, 20-minute timepoint) show visible increases in both Gd and Dy concentration. One additional mouse was serially injected with Dy-DOTA-azide followed by Gd-BOPTA injection 10 minutes later (Supplementary Fig. 2). The delayed increase in Gd concentration suggests that each agent is being independently measured.

### Comparison of DC-MRF and ICP-MS

**Figure 4:**
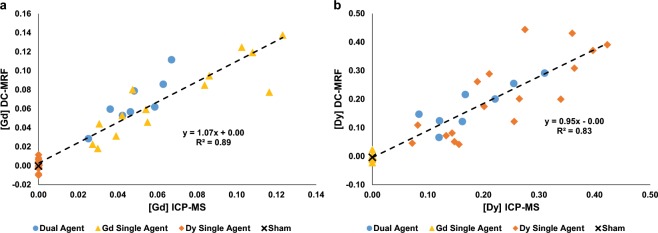
Comparison of DC-MRF and ICP-MS concentration measurements. Tumor (**a**) Gd and (**b**) Dy concentration assessments are plotted against corresponding ICP-MS assessments. These plots include both single agent (n = 14 Gd-only, yellow triangle; n = 17 Dy-only, orange diamond), dual agent (n = 8, blue circle), and sham experiments (n = 1, black ‘x’). Inclusion of all experiments (n = 40) resulted in significant correlations between the DC-MRF and ICP-MS assessments (R^2^ ≥ 0.83, p < 1e^−6^). Subgroup analysis of just the single agent and dual agent experiments also resulted in significant Pearson correlations for both Gd and Dy (R^2^ ≥ 0.64, p < 0.007, Supplementary Fig. 3). All single agent experiments were included to assess the ability of the DC-MRF method to estimate concentration in the extreme case of one agent being absent from the sample. Note that this results in several “zero” points for each single agent experiment (i.e., Gd concentration is approximately zero for Dy-only experiments and Dy concentration is approximately zero for Gd-only experiments).

**Figure 5:**
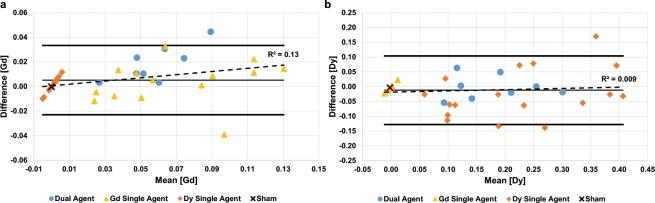
Bland-Altman plots comparing the DC-MRF and ICP-MS assessments of tumor (**a**) Gd and (**b**) Dy concentration. These plots include both single agent (n = 14 Gd-only, yellow triangle; n = 17 Dy-only, orange diamond), dual agent (n = 8, blue circle), and sham experiments (n = 1, black ‘x’). Similar to Fig. 4, all single agent experiments were included to assess the agreement between DC-MRF and ICP-MS in the extreme case of one agent being absent from the sample. Confidence intervals (mean ± 2 standard deviations) are included in each plot along with the mean difference and a linear regression line (dashed line) for all experiments (n = 40). These plots show only 2/40 points (Gd) and 3/40 points (Dy) outside of the 95% confidence intervals. A significant trend was observed for the Gd concentration estimates for both the dual agent experiments (n = 8, p = 0.02) and with all experiments combined (n = 40, p = 0.02). No other assessment showed a significant trend. (Supplementary Fig. 4, Table 1).

Pearson correlations and Bland-Altman plots were used to compare the *in vivo* DC-MRF findings with gold standard ICP-MS elemental analyses (Figs. 4 and 5, respectively). The mean tumor Gd and Dy concentrations were compared for all *in vivo* experiments (n = 40 total) including the dual agent experiments (n = 8, blue circles in Figs. 4 and 5), Gd single agent experiments (n = 14, yellow triangles in Figs. 4 and 5), Dy single agent experiments (n = 17, orange diamonds in Figs. 4 and 5), and sham experiment (n = 1, black ‘x’ in Figs. 4 and 5). Inclusion of all experiments allowed the DC-MRF findings to be compared with gold-standard ICP-MS measurements over a range of tumor concentrations and experimental conditions including the extreme case of one agent not being present in the tumor. Note that the DC-MRF method estimates the Dy concentration near zero for the Gd-BOPTA single agent experiments and the Gd concentration near zero for the Dy-DOTA-azide single agent experiments as appropriate. Overall, both the mean tumor Gd (R^2^ = 0.89, p < 1e^−6^, n = 40) and Dy (R^2^ = 0.83, p < 1e^−6^, n = 40) measurements obtained from the DC-MRF methodology resulted in significant Pearson correlations with the ICP-MS results when all experiments were included (Fig. 4).

Subgroup analyses were also performed on the single and dual agent experiments **(**Supplementary Figs. 3 and 4**)** with Table 1 summarizing the results of the statistical analyses. These subgroup analyses revealed significant Pearson correlations for both the Gd and Dy concentration assessments between DC-MRF and ICP-MS measurements for both the single agent and dual agent studies (Table 1). These initial *in vivo* DC-MRF findings also revealed an overestimation of the Gd concentration (slope of the linear fit = 1.54) and an underestimation of the Dy concentration (slope of the linear fit = 0.85) in the dual agent studies (Supplementary Fig. 3). These slopes are in contrast to the single agent findings where the slopes were close to one (Gd: slope = 0.99, Dy: slope = 1.02) suggesting limited bias in the dual agent studies discussed further below.

**Table 1:** Comparison of DC-MRF and ICP-MS Concentration Measurements.

Gd Measurement	All Studies (n = 40)	Single Agent Only (n = 14)	Dual Agent Only (n = 8)
**Intra-Class Correlation**			
ICC	0.94	0.92	0.40
**Pearson Correlation (DC-MRF vs ICP-MS)**			
R^2^	0.89	0.79	0.76
p-value	<1e-6	0.00002	0.005
**Bland-Altman Analysis (DC-MRF vs ICP-MS)**			
R^2^	0.13	0.05	0.60
p-value	0.02	0.45	0.02
Dy Measurement	All Studies (n = 40)	Single Agent Only (n = 17)	Dual Agent Only (n = 8)
**Intra-Class Correlation**			
ICC	0.90	0.78	0.88
**Pearson Correlation (DC-MRF vs ICP-MS)**			
R^2^	0.83	0.64	0.73
p-value	<1e-6	0.0001	0.007
**Bland-Altman Analysis (DC-MRF vs ICP-MS)**			
R^2^	0.009	0.15	0.0002
p-value	0.56	0.13	0.97

Summary of statistical comparison between DC-MRF and ICP-MS concentration. Dy and Gd analyses are presented separately. R^2^ values represent Pearson Correlations.

Bland-Altman plots and intra-class correlation coefficients (ICCs) were also prepared to test for agreement between the DC-MRF assessments of tumor Gd and Dy concentration and the ICP-MS findings (Fig. 5, Table 1, Supplementary Fig. 4). For the combined single agent and dual agent results (n = 40, Fig. 5) only two and three differences between DC-MRF and ICP-MS out of 40 total scans were outside of the 95% confidence interval (mean ± 2 standard deviations) for the Gd and Dy concentration assessments, respectively. A significant trend in the Bland-Altman plots was observed for the Gd concentration estimates (p = 0.02) but not for the Dy concentration estimates (p = 0.56). Similarly, for the dual agent studies, a significant trend was observed for the Gd concentration estimates (p = 0.02) but not the Dy concentration estimates (p = 0.97) (Supplementary Fig. 4c,d). No significant trends were observed in the Bland Altman plots for the single agent studies (Supplementary Fig. 4a,b).

ICCs confirmed reasonable agreement between the DC-MRF and ICP-MS assessments for both agents when analyzing all experiments together (n = 40; Gd: ICC = 0.94, Dy: ICC = 0.90). Reasonable agreement was also observed for the single agent studies (Gd: n = 14, ICC = 0.92; Dy: n = 17, ICC = 0.78) and the Dy concentration assessments from the dual agent studies (ICC = 0.88, n = 8). Only the Gd concentrations from the dual agent studies showed a substantially diminished agreement (ICC = 0.40, n = 8) despite a significant correlation (R^2^ = 0.76, p = 0.005).

### Subset analysis of *in vivo* relaxivity and DC-MRF concentration estimates

A subset analysis of the single agent and dual agent MRF studies was also conducted to evaluate the stability and reproducibility of the contrast agent quantification. This subset analysis utilized 10 unique randomized subsets of the single agent experiments to provide 10 different estimates of the *in vivo* Gd and Dy relaxivities that were, in turn, used to calculate the DC-MRF Gd and Dy concentrations for the remaining single agent (n = 4 for Gd-only experiments, n = 7 for Dy-only experiments) as well as the dual agent experiments (n = 8). These Gd and Dy concentrations (n = 19 total: n = 8 dual agent and n = 11 single agent) were then compared with the ICP-MS findings for each of the 10 subsets. The resulting *in vivo* Gd and Dy relaxivity estimates as well as the Pearson correlation coefficients and slopes for the MRF and ICP-MS comparisons for each run of the subset analysis and are shown in Supplementary Table 1. Overall, estimates of the *in vivo* Gd and Dy relaxivities exhibited 10–16% variation across the 10 subset analyses (standard deviation/mean value *100%). All of the subset analyses resulted in significant linear correlations between the DC-MRF and ICP-MS concentrations (Gd Pearson Correlation Coefficient Range = 0.87–0.96; Dy Pearson Correlation Coefficient Range = 0.83–0.95) with only 3–4% variation observed in the correlation coefficients. The mean slope of the correlations between MRF and ICP-MS were 1.13 and 0.94 for Gd and Dy, respectively. These correlation slopes exhibited 18–21% variation, suggesting that individual experiments may be significantly impacting the comparison between the DC-MRF and ICP-MS findings. These results also suggest substantial bias between the ICP-MS and DC-MRF findings.

## Discussion

Overall, these initial *in vivo* results show that dynamic quantitative MRI assessments via MRF combined with the multi-agent relaxation model (Eqs. 2a and 2b) provides a non-invasive platform to simultaneously monitor two paramagnetic MRI contrast agents. While the DC-MRF method could ultimately have numerous clinical and preclinical imaging applications, a particularly attractive application stems from the recent development of MRI biosensors to detect changes in pH^18–20^, neurotransmitter release^8^, and other important biological features^11,21^. Combining the active sensor (Agent A) with an on-board control (inactive sensor, Agent B) would enable estimation of absolute sensor activity (absolute pH, neurotransmitter concentration level, etc.) instead of tracking only relative differences that can be prone to misinterpretation, especially during eventual translational imaging studies in human diseases with known spatial and/or temporal variation. In this way, DC-MRF provides a practical methodology for quantitative molecular MRI studies with built in controls similar to those used in almost all basic science experiments.

A key feature of the DC-MRF methodology is the flexibility in implementation. While this initial *in vivo* study was conducted with a primarily T_1_ contrast agent (Gd-BOPTA) and a primarily T_2_ contrast agent (Dy-DOTA-azide), a wide variety of impactful contrast agent combinations could be identified. The only requirement for the selected contrast agents is that they have sufficiently different relaxivity ratios (r_2_/r_1_)^22–24^ allowing Eqs. 2a and 2b to be solved analytically. Further, the advantage of the MRF acquisition itself is that it has already been shown to provide simultaneous and repeatable measurements of T_1_ and T_2_ relaxation time constants in non-contrast studies on both animal^15,25–27^ and human MRI scanners^28–31^ spanning a wide range of field strengths with improved precision and temporal efficiency in comparison to conventional quantitative MRI techniques. MRF has also shown the ability to measure numerous MRI tissue properties beyond T_1_ and T_2_^32–36^. This ability to quantify a wide range of MRI tissue properties suggests that true “multi-color” MRI using three or more contrast agents may also be possible. Therefore, while virtually any multi-parametric MRI method could be used to detect two MRI contrast agents using the multi-agent relaxation model, MRF provides a repeatable, dynamic imaging platform that could also eventually be expanded to assess more than two contrast agents.

A major component of the DC-MRF methodology is the multi-agent relaxation model (Eqs. 2a and 2b). Using these equations to solve for contrast agent concentration requires accurate estimation of the *in vivo* magnetic relaxivities (r_1_ and r_2_) for each contrast agent. Because of the analytical solution, errors in the magnetic relaxivity estimates will correspondingly propagate to errors in the calculated contrast agent concentrations. This is particularly important for *in vivo* applications where relaxivity values, especially r_2_, can change substantially based on the local molecular environment^37^. It is critical to note that the estimated magnetic relaxivities may also be influenced by the acquisition methodology. In this study, the FISP-MRF imaging kernel incorporated variation in the echo time, repetition time, and flip angle likely adding sensitivity to B_0_ inhomogeneities and T_2_* relaxation to the MRF signal, images, and corresponding T_1_ and T_2_ maps^35,36^. It is possible that these additional sensitivities may also contribute to the observed differences between the *in vivo* and *in vitro* relaxivities. In this study, the *in vivo* and *in vitro* r_1_ values were relatively consistent (Gd,Dy r_1_: 5.63/0.25 *in vivo* vs 5.33/0.16 *in vitro*), but we observed substantial differences between the *in vitro* and *in vivo* r_2_ estimates (Gd, Dy r_2_: 37.08/93.59 *in vivo* vs 6.73/6.02 *in vitro*). While the underlying mechanism for differences in r_2_ relaxivity remain uncertain, these results stress the importance of obtaining accurate estimates of the *in vivo* magnetic relaxivities in order to obtain reasonable concentration estimates from the multi-agent relaxation model. As such, in this study, we completed numerous single agent experiments (Gd: n = 14; Dy: n = 17), including a sham control study (n = 1), to obtain reasonable estimates for the *in vivo* magnetic relaxivities before performing the dual agent experiments. However, the subset analyses revealed a 10–16% variation in the *in vivo* relaxivities across the different subsets (Supplementary Table 1), further highlighting the challenge and importance of obtaining accurate *in vivo* relaxivity estimates.

Despite its previously-described improvements in acquisition efficiency and precision, MRF-based quantification schemes are susceptible to acquisition imperfections and dictionary simulation assumptions that can lead to errors in the MRF-based T_1_ and T_2_ measurements. For example, prior studies have shown that T_2_ measurements using MRF can be impacted by B_1_^+^ field heterogeneity^27,38^. Other potential T_1_ and T_2_ error sources include the assumption of mono-exponential relaxation in the MRF simulations as well as T_2_*/off-resonance effects^35,36^ that are enhanced *in vivo*. For the DC-MRF methodology, these errors in either absolute T_1_ and T_2_ and/or the changes in T_1_ and T_2_ can lead to downstream errors in the Gd and Dy concentration estimates. These error sources may be a significant factor in the observed difference between the DC-MRF Gd and Dy concentrations in comparison to ICP-MS for both the dual agent studies (Supplementary Fig. 3) as well as the subset analysis (Supplementary Table 1). Future *in vivo* studies are needed to help elucidate the impact of paramagnetic contrast agents and these error sources on the DC-MRF concentration estimates.

A key next step in the development of the DC-MRF methodology will be to extend the dynamic MRF acquisition to three dimensions to minimize the effect of tissue heterogeneity when comparing DC-MRF and ICP-MS measurements and better capture the anatomy and physiology of the entire tumor. Currently, limitations of this study include the assumptions that: (1) the imaging slices used in the ROI analysis were representative of the entire 3D tumor; and (2) mean Gd and Dy concentrations within the imaging slice calculated from the ROI analysis were representative of the actual tumor concentrations despite expected spatial variation. Therefore, imaging of a non-representative slice may also have caused some of the discrepancies seen between the DC-MRF and ICP-MS measurements. A true 3D dynamic DC-MRF acquisition would eliminate these assumptions and possibly improve the accuracy of the DC-MRF measurements, which would be particularly valuable when studying patient-derived xenograft models that more closely mimic heterogeneous human tumors. This 3D approach may be possible using highly undersampled non-Cartesian k-space trajectories^39^ as used in this study, and would also provide the increased SNR and/or improved spatial resolution needed for some preclinical MRF studies. However, a true 3D MRF acquisition may also lack the temporal resolution needed to dynamically monitor some MRI contrast agents *in vivo*. An alternative approach would be to use an interleaved multi-slice MRF acquisition that would provide concentration maps over multiple slices for nearly the same acquisition time as presented herein^30^. Regardless, additional *in vivo* experiments with a multi-slice and/or 3D DC-MRF methodology will be needed to more thoroughly evaluate the accuracy of the DC-MRF methodology in comparison to elemental analyses.

In conclusion, these results describe the first *in vivo* implementation of the DC-MRF methodology. These initial *in vivo* results demonstrate that DC-MRF and the multi-agent relaxation model can dynamically estimate the concentration of two paramagnetic MRI contrast agents (i.e., Gd and Dy chelates) over a range of concentrations. Further, these DC-MRF concentration assessments resulted in significant linear correlations with gold-standard elemental analysis of tissue specimens. Importantly, DC-MRF may have numerous clinical and preclinical imaging applications as this methodology can be readily adapted to assess a wide variety of diseases using conventional and/or newer molecular MRI contrast agents. Future work will need to explore more efficient mechanisms to estimate the *in vivo* relaxivities, perform *in vivo* voxelwise assessments of multiple contrast agents, and further refine the MRF acquisition (e.g., multi-slice or 3D MRF) to provide a more direct comparison with elemental analyses.

## Materials and Methods

### Mouse glioma model

All animal studies were conducted according to protocols approved by the Institutional Animal Care and Use Committee (IACUC) at Case Western Reserve University. The heterotopic glioma tumor model used has been described in detail previously^40^. Briefly, 2 × 10^6^ green-fluorescent protein (GFP)-expressing human LN-229 cells were mixed with Matrigel Matrix (BD Biosciences, Franklin Lakes, NJ, USA) and injected into the right flank of female NIH athymic nude female mice bred in the Athymic Animal Core Facility at Case Western Reserve University. Mice with flank tumor diameters ranging from 5–10 mm were then selected for imaging (typically 4–7 weeks post-implantation).

### Preparation and characterization of Dy-DOTA-azide

DOTA-azide (Macrocyclics, Inc., Plano, TX, USA) was dissolved in water with 1.2 equivalents of DyCl_3_•6H_2_O (Strem Chemicals, Newburyport, MA, USA). The pH of the solution was adjusted to ~5.0 with 1.0 M NaOH and the reaction mixture was stirred at room temperature for 24 hours. The pH was monitored every 4 hours and adjusted as needed with additional NaOH to maintain a pH of about 5.0. Once Dy-DOTA-azide synthesis was complete, the pH was raised to 7.0 to precipitate any free Dy, centrifuged, and lyophilized to yield a hygroscopic white powder^23^. The molecular weight of the Dy-DOTA-azide complex was confirmed by matrix-assisted laser desorption/ionization time-of-flight (MALDI-TOF; Autoflex Speed, Bruker Corp., Billerica, MA, USA) as shown in Supplementary Fig. 5.

### *In vitro* MRI characterization of Gd-BOPTA and Dy-DOTA-azide

*In vitro* relaxivities of Gd-BOPTA and Dy-DOTA-azide were measured at 9.4 T using phantoms containing serial dilutions of each agent with 0.9% saline (Fisher Scientific, Fairlawn, NJ, USA) in 5 mm NMR tubes (Norell, Morganton, NC, USA) (Gd-BOPTA phantoms at 0.05, 0.1, 0.2, and 0.3 mM; Dy-DOTA-azide phantoms at 2.0, 4.0, 6.0, and 8.0 mM; pure saline phantom included as control). Phantom T_1_ relaxation time constants were measured using a single echo inversion recovery spin echo sequence with a repetition time of 10,000 ms, an echo time of 8 ms, and inversion times of 50, 500, 1000, 2500, 4500, and 9000 ms. T_2_ relaxation time constants were measured using a CPMG multi-echo spin echo method with echoes measured every 25 ms for 20 echoes. Relaxation time constants were estimated using a least-squares fit to the appropriate exponential recovery model. Mean T_1_ and T_2_ values for all the phantoms were plotted against the concentration of the agent and a linear regression was performed to estimate the r_1_ and r_2_ relaxivities of each agent. The *in vitro* relaxivity values were found to be: r_1_ = 5.33 and 0.16 L/mmol/sec and r_2_ = 6.73 and 6.02 L/mmol/sec at 9.4 T for Gd-BOPTA and Dy-DOTA-azide, respectively (Supplementary Fig. 1).

### Dynamic spiral MRF acquisition design and dictionary preparation

All MRI experiments were performed on a Bruker Biospec 9.4 T MRI scanner. A rapid MRF acquisition was implemented using a Fast Imaging with Steady-state Free Precession (FISP) imaging kernel and a variable-density, undersampled spiral trajectory as described previously^15,25^. Each MRF acquisition consisted of an initial adiabatic inversion pulse followed by 1024 FISP imaging kernels (MRF imaging timepoints) with varying repetition times (range = 9.5 to 12.2 ms) and flip angles (range = 0 to 60 degrees). The echo time (TE) for each imaging kernel was set as TR/2. The FISP imaging kernel also incorporated a spoiler gradient following each spiral acquisition to limit banding artifacts associated with TrueFISP acquisitions on high field MRI scanners^25^.

The variable-density spiral trajectory was designed to fully sample k-space with 48 interleaves, have an imaging field of view of 3 cm × 3 cm, and a regridded Cartesian matrix of 128 × 128. All Images were reconstructed using the non-uniform Fast Fourier Transform reconstruction toolbox^41^. Each spiral interleaf acquired 1000 data points with a readout time of 5 ms. Sequential interleaves were rotated by an angle of 7.5° in order to distribute the interleaves across k-space^15^. A 9-second delay was incorporated after each dynamic MRF series to limit the duty cycle on the magnetic field gradients and to allow the magnetization to recover to equilibrium prior to starting the next MRF series. This design resulted in an acquisition time of 21 seconds to obtain one spiral interleaf for 1024 images. For these dynamic *in vivo* studies, 6 spiral interleaves were acquired for each MRF scan (reduction factor = 8) resulting in a total acquisition time of 126 seconds to acquire a single set of T_1_ and T_2_ maps.

The MRF dictionary for the FISP-MRF acquisition was generated by simulating the Bloch equations using a FISP sequence with the imaging parameters described above and every logical combination of T_1_ and T_2_ within a specified range. Mono-exponential relaxation was assumed during Bloch equation simulation similar to prior MRF studies which showed reasonable agreement for T_1_ and T_2_ values between MRF and conventional MRI techniques^26,28,29^. The simulated T_1_ relaxation time constant was varied from 50–10,000 ms (50–3000 ms, increment = 10 ms; 3000–5000 ms, increment = 100 ms; 5000–10,000 ms, increment = 200 ms), and the simulated T_2_ relaxation time constant was varied from 2–800 ms (2–200 ms, increment = 2 ms; 200–500 ms, increment = 10 ms; 500–800 ms, increment = 50 ms) resulting in 44,667 dictionary entries. The simulated MRF dictionary was then used to generate the T_1_ and T_2_ maps for each acquired MRF dataset by comparing the acquired MRF signal evolution profile from each voxel to all of the simulated signal evolution profiles in the MRF dictionary using an inner product formalism described previously^29^.

### *In vivo* DC-MRF assessments

Tumor-bearing mice (n = 40) were anesthetized with 2% isoflurane in 100% oxygen (1.0 L/min), and a 26-gauge catheter (Covidien, Mansfield, MA, USA) was placed in a tail vein to administer a bolus of the contrast agents as described previously^42^. Injected doses for all studies were based on individual mouse weights and delivered in a total volume of 150 μL over 90 seconds. Animals were placed in the right lateral decubitus position within a 35-mm birdcage volume coil in the 9.4 T MRI scanner. Respiration rate (40–60 breaths per minute) and core body temperature (35 ± 1 °C) were maintained by adjusting the isoflurane level and warm air, respectively.

Conventional localizer scans were first acquired to position the imaging slice in the center of each flank tumor for the axial single-slice dynamic MRF acquisition. Automated localized shimming over the imaging slice was performed using a conventional ^1^H PRESS MRS acquisition^43^ prior to the MRF scans to minimize off-resonance effects. All MRF acquisitions used the undersampled spiral FISP-MRF acquisition described above resulting in T_1_ and T_2_ relaxation time constant maps (3.0 × 3.0 cm FOV, 128 × 128 matrix, slice thickness = 1.5 mm) every ~2 minutes. Pre-contrast assessments of tumor T_1_ and T_2_ (tumor T_10_ and T_20_) were made using the ten MRF scans acquired prior to injecting any agent. At the beginning of the 11^th^ MRF scan, the agent was injected. A total of 10 post-contrast MRF scans (20 total dynamic MRF scans) were completed for each animal to assess contrast agent dynamics.

All acquired MRF data was exported to MATLAB (MathWorks, Natick, MA, USA) for analysis. Raw MRF images were generated by using established regridding and density compensation procedures described previously. The raw MRF signal profiles from each individual voxel were matched to the same simulated dictionary for each of the 20 acquired MRF scans. The result was 20 T_1_ and T_2_ maps with a temporal separation of ~2 minutes.

After acquisition of the final MRF dataset, the mouse was immediately euthanized via cervical dislocation and its tumor excised and weighed. The tumor was placed in a glass vial and an equal volume of trace metals grade Nitric Acid (Fisher Scientific, Fair Lawn, NJ, USA) was added based on total tumor mass. Samples were vortexed once a day for 7 days and the resulting volume of the liquefied sample was determined before centrifugation for 15 min at 20,000 RPM. Samples were diluted at least 20-fold in ultrapure water based on the volume recovered from the digestion, and filtered through 0.22 micron filters before analysis using a Thermo Scientific XSeries 2 ICP-MS (Thermo Fisher Scientific, Bremen, Germany). Gd and Dy content in tumors was determined via inductively coupled plasma-mass spectrometry (ICP-MS) on samples prepared and sent to the Center for Materials and Sensor Characterization, University of Toledo, Toledo, OH, USA.

### DC-MRF assessment of tumor gd and dy concentration in a mouse glioma model

In the first set of dynamic MRF experiments, the Gd and Dy MRI contrast agents were administered individually in order to estimate the intra-tumoral magnetic relaxivities (r_1_ and r_2_) for each MRI contrast agent. For the *in vivo* relaxivity assessments of the Gd contrast agent, 14 mice were manually injected with a bolus of 0.1–0.4 mmol/kg of Gd-BOPTA. For the *in vivo* relaxivity assessments of the Dy contrast agent, 17 mice were manually injected with a bolus of 0.3–1.3 mmol/L of Dy-DOTA-azide. A single mouse was injected with 150 uL of saline as a sham control. These concentration ranges were selected to provide both T_1_ and T_2_ contrast sensitivity for each MRI contrast agent enabling estimates of r_1_ and r_2_ for each contrast agent.

All MRI data used for comparison with ICP-MS measurements were analyzed using a region-of-interest (ROI) analysis. The ROIs for each experiment were drawn on an anatomical reference scan acquired immediately prior to starting the dynamic DC-MRF acquisition. The entire tumor area was selected as shown in Supplementary Fig. 6, unless major artifacts (i.e., susceptibility, motion) were observed. The ROI analysis of MRF-based T_1_ and T_2_ maps generated mean tumor T_1_ and T_2_ values for each mouse at each dynamic MRF experiment with tumor T_10_ and T_20_ values for Eqs. 1a and 1b estimated by calculating the mean tumor T_1_ and T_2_ for the 10 pre-contrast MRF scans. Mean tumor T_1_ and T_2_ values from the final post-contrast MRF scan (scan #20) were used as estimates of T_1_ and T_2_ in Eqs. 1a and 1b, respectively. Measured tumor Gd and Dy concentrations were obtained from the ICP-MS elemental analysis. Using these values, a linear regression of ΔR_1_ (1/T_1_–1/T_10_) and ΔR_2_ (1/T_2_–1/T_20_) as a function of Gd and Dy concentration was used to estimate tumor r_1_ and r_2_ for each MRI contrast agent^13^.

After estimation of the tumor relaxivities for the Gd and Dy contrast agents, dynamic MRF scans were then obtained for 8 mice simultaneously injected with a mixed solution containing both the Gd and Dy MRI contrast agents. The injected Gd (0.15–0.30 mmol/kg) and Dy (0.30–1.10 mmol/kg) doses were varied to observe a range of T_1_ and T_2_ enhancement and to compare the DC-MRF findings with ICP-MS over a range of agent concentrations. Similar to the single agent studies, 10 pre-contrast MRI scans and 10 post-contrast MRF scans were performed for each mouse, and MRF-based T_1_ and T_2_ maps were generated for each of the 20 total MRF scans. Using the *in vivo* relaxivities estimated from the single agent studies, the MRF-based T_1_ and T_2_ maps were used to calculate voxelwise Gd and Dy concentration maps for each animal and each MRF scan by directly solving Eqs. 2a and 2b ^14^. Tumor Gd and Dy maps were generated for all single agent and dual agent studies (n = 40). The ROI analysis of these maps generated mean tumoral Gd and Dy concentration curves as a function of time for each animal. The mean tumor Gd and Dy concentration obtained from the final post-contrast MRF scan (Scan 20) was compared to the corresponding concentration measured via elemental analysis for all mice (n = 40 total mice for single agent, dual agent, and sham experiments).

Additionally, a single mouse was injected with Dy-DOTA-azide at the beginning of scan 11, followed by injection of Gd-BOPTA at the beginning of scan 15 (10-minute delay). Each bolus for this sequential experiment was delivered in a total of 75 μL over 60 seconds. The MRI data for this mouse was processed in the same way as the other two agent experiments but was not included in the ICP-MS analyses.

### Comparison of relaxivity and concentration estimates from randomized subsets

A subset analysis of the single agent and dual agent data was conducted to assess the stability of the relaxivity measurements and subsequent accuracy of the concentration estimates. In this subset analysis, the *in vivo* relaxivities were estimated from Eqs. 1a and 1b as described above, using a randomly selected subset of the single agent experiments (10 Gd-only and 10 Dy-only single agent experiments) in combination with the sham experiment (no contrast agent). The remaining single agent studies (n = 4 for Gd-only studies; n = 7 for Dy-only studies) were then combined with the dual agent studies (n = 8) to compare the DC-MRF concentration estimates with the ICP-MS findings. This selection process ensured no overlap of experiments used to estimate the *in vivo* relaxivities and the comparison between the ICP-MS and DC-MRF tumor concentrations. The subset analysis was repeated 10 times with a unique combination of randomly selected sets of single agent experiments using a MATLAB randomization function.

### Statistics

Pearson correlations were used to test for significant correlations between the MRF-based changes in T_1_ and T_2_ relaxation time constants with ICP-MS assessments of tumor Gd and Dy concentration to obtain estimates of *in vivo* magnetic relaxivities (r_1_ and r_2_) (Fig. 2). Pearson correlations were also used to test for significant correlations between the DC-MRF and ICP-MS assessments of tumor Gd and Dy concentration (Fig. 4, Supplementary Fig. 3). Intra-class correlations (ICC) and Bland-Altman plots (Fig. 5, Supplementary Fig. 4) were used to test for agreement between the DC-MRF and ICP-MS results. Pearson correlations were also used to test for significant trends in the Bland-Altman plots (Fig. 5). For all statistical tests, subgroup analyses were performed on the single agent and dual agent experiments with all results summarized in Table 1. A significance level, α, of 0.05 was used to test for significance in all associations.

## Supplementary information

Supplementary Info

## Data Availability

Raw data from the dynamic MRF assessments and the corresponding ICP-MS results for all *in vivo* imaging experiments will be made available by the corresponding author upon request to evaluate these *in vivo* DC-MRF results. MRF dictionaries and MATLAB code will also be made available upon reasonable request to reproduce and expand upon these MRI findings.
