# Supplementary material for: Dynamic, Simultaneous Concentration Mapping of Multiple MRI Contrast Agents with Dual Contrast - Magnetic Resonance Fingerprinting
Source: Sci Rep. 2019 Dec 27;9:19888. doi: 10.1038/s41598-019-56531-7 (PMC6934650; doi:10.1038/s41598-019-56531-7)
Supplement: Supplementary file 1 — Supplementary Info [file 41598_2019_56531_MOESM1_ESM.pdf]

Dynamic, Simultaneous Concentration Mapping of Multiple MRI Contrast Agents with Dual Contrast - Magnetic Resonance Fingerprinting

Christian E. Anderson[1,2], Mette Johansen[3], Bernadette O. Erokwu[1], He Hu[2,4], Yuning Gu[2], Yifan Zhang[1], Michael Kavran[1], Jason Vincent[3], Mitchell L. Drumm[5,6], Mark A. Griswold[1,2], Nicole F. Steinmetz[1,2,4,7,8], Ming Li[9], Heather Clark[10-12], Rebecca J. Darrah[5,13], Xin Yu[2,14], Susann M. Brady-Kalnay[3,15], Chris A. Flask[1,2,6*]

[1]Department of Radiology, Case Western Reserve University, Cleveland, OH, USA

[2]Department of Biomedical Engineering, Case Western Reserve University, Cleveland, OH, USA

[3]Department of Molecular Biology and Microbiology, Case Western Reserve University, Cleveland, OH, USA

[4]Department of NanoEngineering, University of California-San Diego, La Jolla, CA, USA

[5]Department of Genetics and Genome Sciences, Case Western Reserve University, Cleveland, OH, USA

[6]Department of Pediatrics, Case Western Reserve University, Cleveland, OH, USA

[7]Department of Radiology, University of California-San Diego, La Jolla, CA, USA

[8]Moores Cancer Center, University of California-San Diego, La Jolla, CA, USA

[9]Department of Population and Quantitative Health Sciences, Case Western Reserve University, Cleveland, OH, USA

[10]Department of Bioengineering, Northeastern University, Boston MA, USA

[11]Department of Chemistry and Chemical Biology, Northeastern University, Boston MA, USA

[12]Institute of Systems Bioanalysis and Chemical Imaging, Northeastern University, Boston MA, USA

[13]Francis Payne Bolton School of Nursing, Case Western Reserve University, Cleveland, OH, USA

[14]Department of Physiology and Biophysics, Case Western Reserve University, Cleveland, OH, USA

[15]Department of Neurosciences, Case Western Reserve University, Cleveland, OH, USA

This file contains supplementary figures for the above referenced manuscript to provide additional information with regards to the methods and results presented.

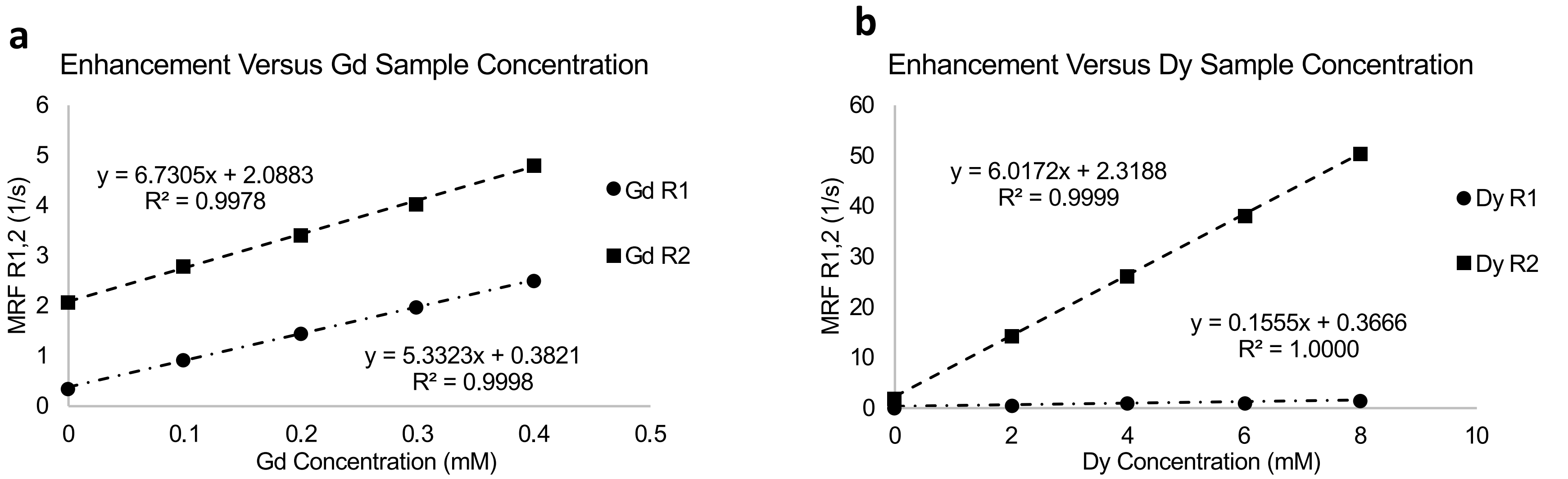

Supplementary Figure 1. In vitro magnetic relaxivity curves for both **(a)** Gd-BOPTA (n=5) and **(b)** Dy-DOTA-azide (n=5) contrast agents. R1 = 1/T1 and R2 = 1/T2 data obtained from spin echo based T1 and T2 relaxation time are plotted against the corresponding known concentration of Gd or Dy in each phantom. A linear least squares regression resulted in significant correlations for all experiments ($p<0.02$). The slope of the linear fit in each plot is the in vitro magnetic relaxivity (r_1 and r_2) of each contrast agent.

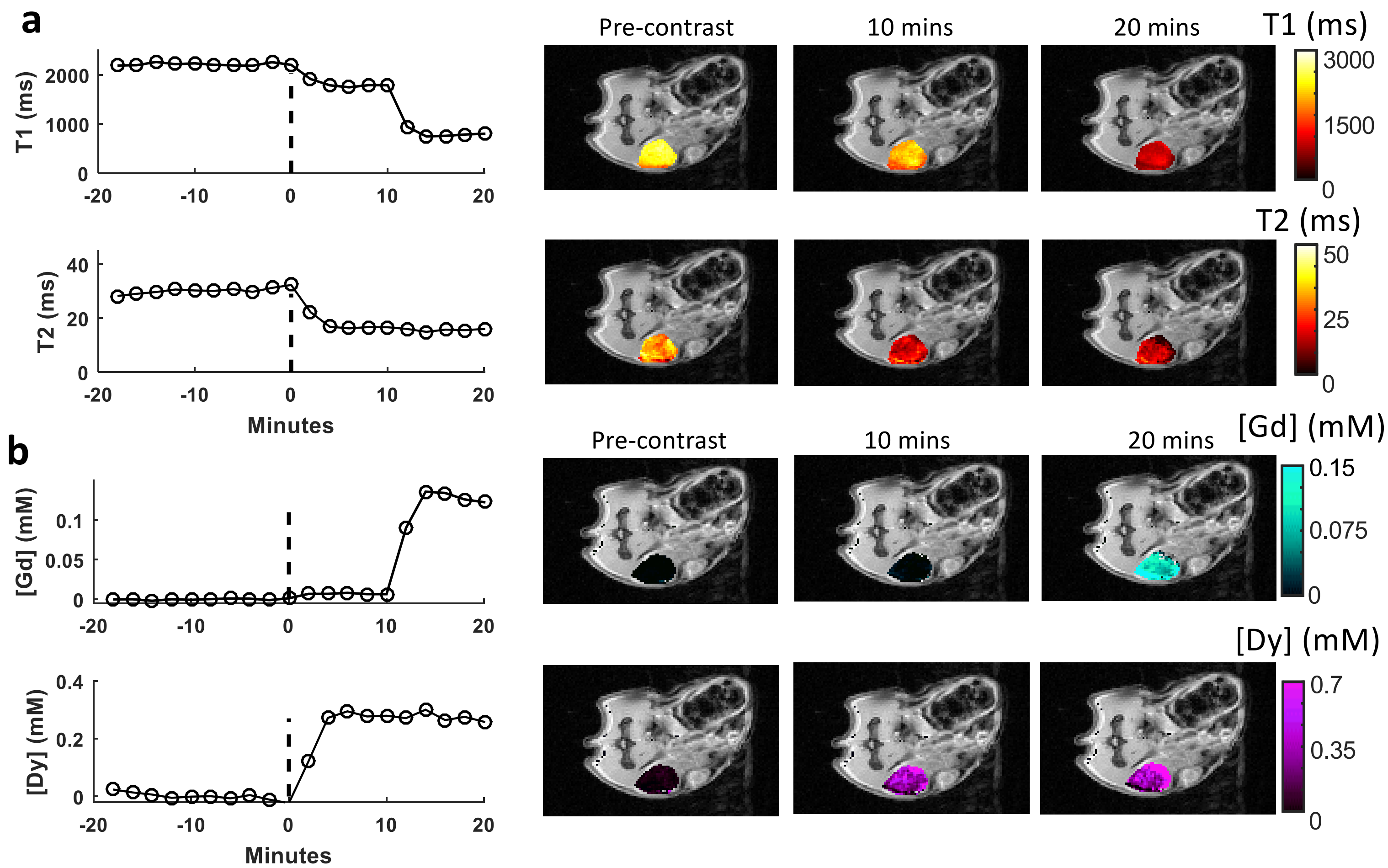

Supplementary Figure 2. (a) T1 and T2 relaxation time curves and maps for sequential bolus injections of the Dy and Gd contrast agents. A bolus of Dy-DOTA-azide was injected at t=0 (dotted line). The bolus of the Gd-BOPTA was injected at t=10 minutes. The magnitude of T1 and T2 changes are in line with expect changes for the high r2 relaxivity of the Dy agent and the high r1 relaxivity of the Gd agent. **(b)** Corresponding tumor Gd and Dy concentration curves for the same experiment. Note the visible increase in Dy concentration after the 0-minute timepoint while the Gd concentration remains near zero concentration until after the 10-minute timepoint.

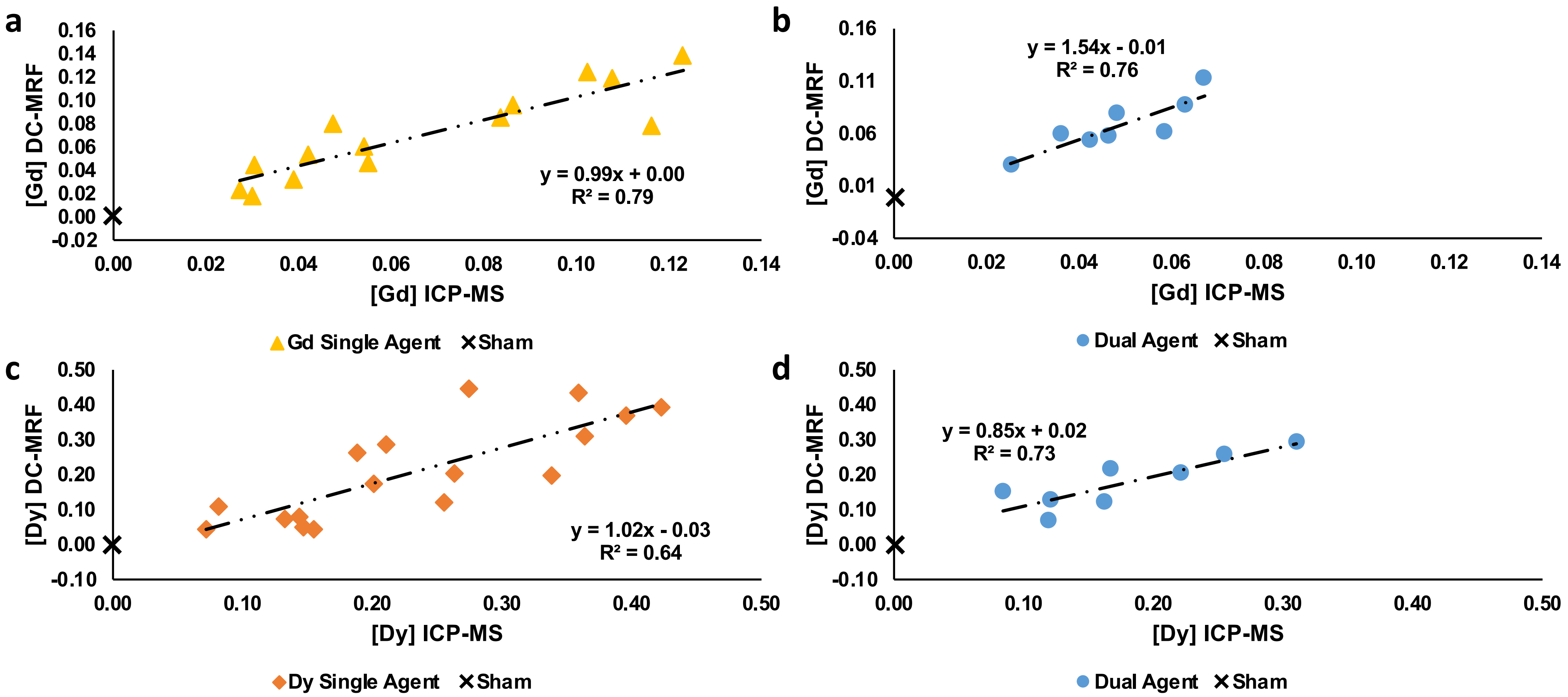

Supplementary Figure 3. Comparison of Pearson correlations between DC-MRF and ICP-MS concentration measurements for mice injected with either **(a)** Gd only (n=14), **(c)** Dy only (n=17), or **(b,d)** both MRI contrast agents simultaneously (n=8). The sham control is shown (black **x**) but is not included in the correlation analyses. All comparisons resulted in significant correlations between the two measurements ($p<0.007$). The dual agent concentration estimates appear to show some bias (slopes = 1.54 and 0.85, respectively) while the single agent studies show limited bias (slopes = 0.99 and 1.02, respectively). The data in these analyses are a subgroup of the data presented in **Figure 4**. Correlation statistics for the plots shown here are listed in **Table 1**.

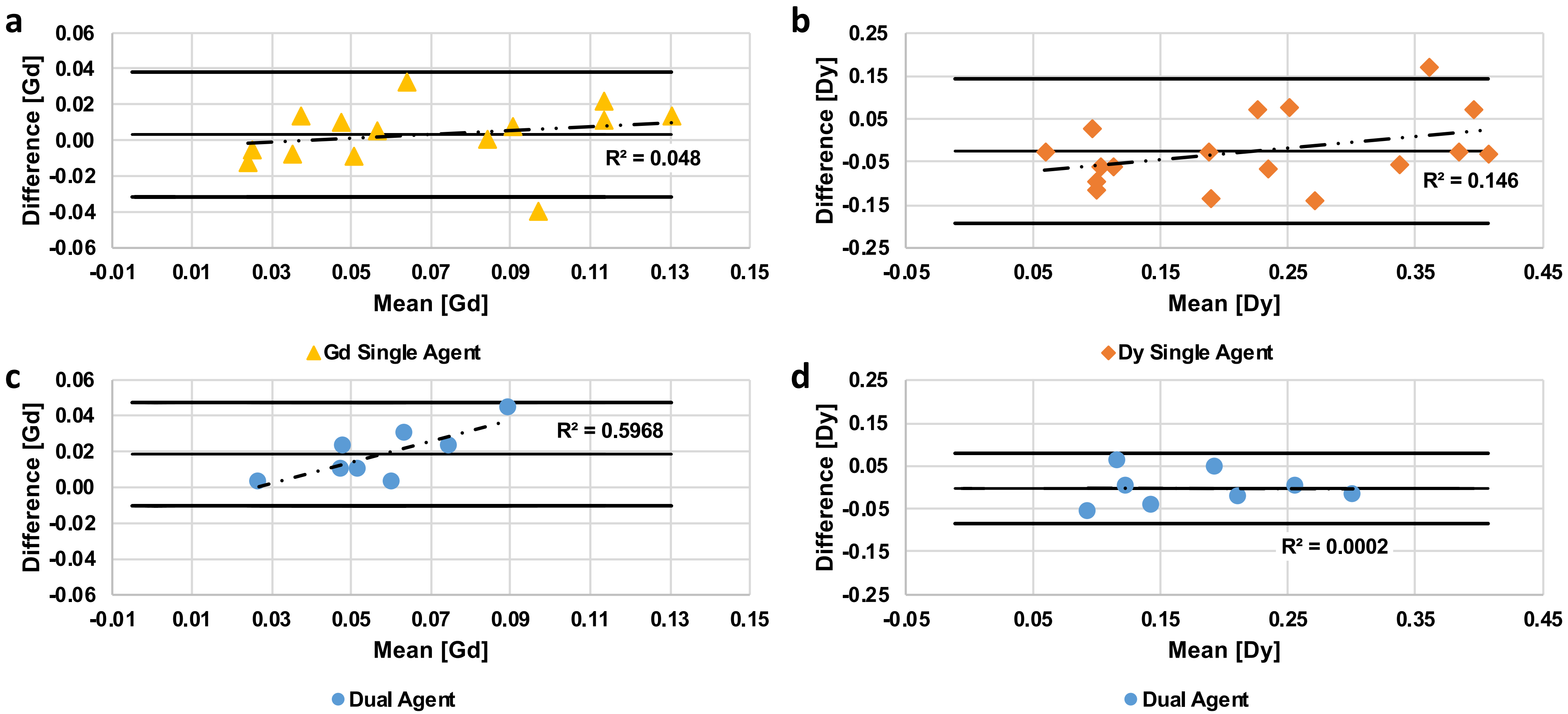

Supplementary Figure 4. Bland-Altman analysis between DC-MRF and ICP-MS concentration measurements for mice injected with either **(a)** Gd only (n=14), **(b)** Dy only (n=17), or **(c,d)** both MRI contrast agents simultaneously (n=8). A significant trend was observed in the Gd estimates obtained for the dual agent studies ($R^2 = 0.60$, $p = 0.02$). No other significant trends were observed. The data in these analyses are a subgroup of the data presented in **Figure 5**.

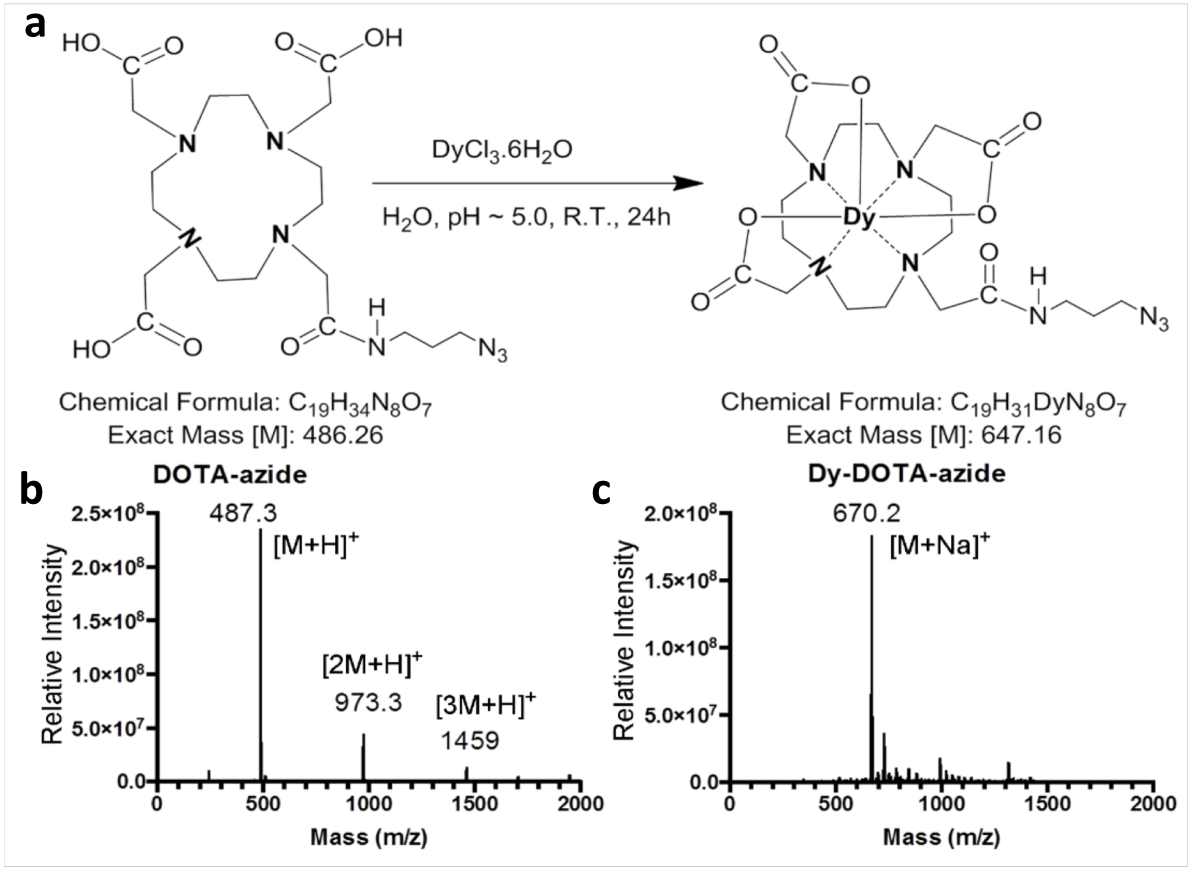

Supplementary Figure 5. (a) Schema showing the synthesis of Dy-DOTA-azide. MALDI-TOF mass spectra of **(b)** DOTA-azide, and **(c)** final Dy-DOTA-azide product.

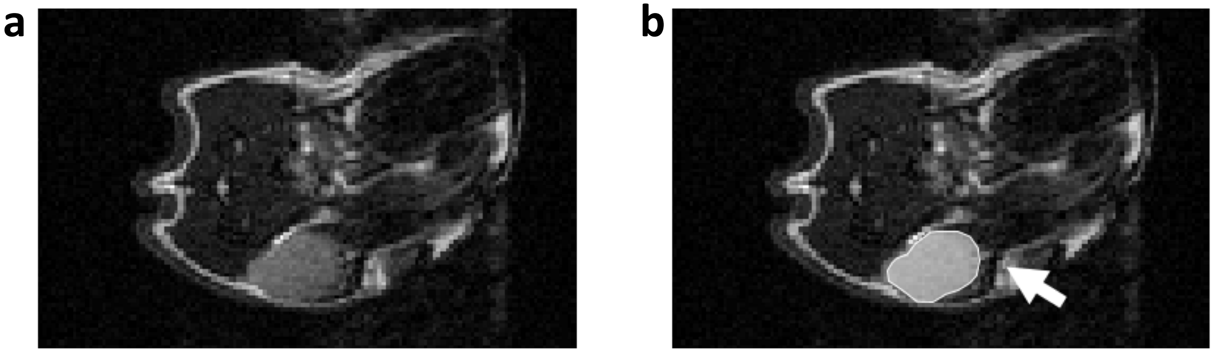

Supplementary Figure 6. Example ROI used for DC-MRF and ICP-MS ROI comparisons. **(a)** Representative gradient echo anatomical reference image acquired prior to MRF mapping to verify animal positioning and image quality. During analysis, this reference image was used to select the ROI used to calculate average T1 and T2 values. **(b)** An example ROI is shown as an overlay on the anatomical reference image. The ROI was selected in a way that incorporated the entire tumor but avoided areas of severe artifact that would corrupt the T1 and T2 measurements (white arrow).

	**Calibration**					**Validation**				
	**Relaxivity**					**Correlations**			**Slope of Correlations**	
**Run**	**r1 Gd**	**r2 Gd**	**r1 Dy**	**r2 Dy**		**R (Gd)**	**R (Dy)**		**Gd**	**Dy**
**1**	7.01	45.97	0.29	94.50		0.89	0.91		0.84	0.93
**2**	6.44	47.42	0.22	106.79		0.90	0.87		0.89	0.78
**3**	5.35	33.09	0.25	94.46		0.96	0.94		1.25	0.88
**4**	6.28	42.99	0.23	108.58		0.87	0.87		0.84	0.67
**5**	5.33	30.69	0.25	96.36		0.95	0.92		1.27	0.87
**6**	6.65	39.71	0.30	88.29		0.91	0.95		0.86	1.01
**7**	5.43	39.54	0.26	88.48		0.96	0.83		1.30	0.92
**8**	5.15	29.25	0.30	77.49		0.96	0.90		1.37	1.31
**9**	5.61	34.51	0.28	91.37		0.95	0.86		1.28	1.00
**10**	5.13	39.49	0.25	90.36		0.96	0.93		1.42	0.98
**Avg**	**5.84**	**38.27**	**0.26**	**93.67**		**0.93**	**0.90**		**1.13**	**0.94**
**Min**	**5.13**	**29.25**	**0.22**	**77.49**		**0.87**	**0.83**		**0.84**	**0.67**
**Max**	**7.01**	**47.42**	**0.30**	**108.58**		**0.96**	**0.95**		**1.42**	**1.31**
**Std. Dev.**	**0.69**	**6.24**	**0.03**	**9.05**		**0.04**	**0.04**		**0.24**	**0.17**
**% Variation**	**11.82**	**16.31**	**10.60**	**9.66**		**3.77**	**4.07**		**21.38**	**18.01**

Supplementary Table 1. Subset analysis of *in vivo* relaxivity estimates and comparison of DC-MRF and ICP-MS findings. Relaxivity values were estimated using unique subsets of the single agent experiments and used to calculate DC-MRF based estimates of Gd and Dy concentration. These DC-MRF concentrations were compared to ICP-MS measurements and their correlations and slope of the correlation are reported for each run. % variation is defined as (standard deviation/average*100).
